# Blood Neutrophil to Lymphocyte Ratio as a Marker of Inflammatory Bowel Disease Severity in Iranian Adults: a Cross-Sectional Survey

**DOI:** 10.34172/mejdd.3261

**Published:** 2026-01-31

**Authors:** Parsia Noori Mirtaheri, Aria Shirani, Mobin Obeidinia, Elham Pishgar

**Affiliations:** ^1^Research Scholar, Medical Physiology Research Center, Iran University of Medical Sciences, Tehran, Iran; ^2^Cardiology Resident, School of Medicine, Tehran University of Medical Sciences, Tehran, Iran; ^3^Research Scholar, Gastrointestinal and Liver Diseases Research Center, Firoozgar Hospital, Iran University of Medical Sciences, Tehran, Iran; ^4^Colorectal Research Center, Iran University of Medical Sciences, Tehran, Iran

**Keywords:** Inflammatory bowel disease, Ulcerative colitis, Crohn disease, Blood neutrophile-to-lymphocyte ratio

## Abstract

**Introduction:**

Given the increase in the prevalence of inflammatory bowel diseases (IBD) in developing countries like Iran, studies to find new markers for disease monitoring seem necessary. Thus, we aimed to evaluate the value of a novel non-invasive marker, the neutrophil-to-lymphocyte ratio (NLR), in assessing the severity and activity of IBD in adults.

**Methods:**

We collected retrospective data from adult patients with a history of ulcerative colitis (UC) or Crohn’s disease (CD) who attended the clinic, gathering information from one month prior to their enrollment. The data included demographics, the Mayo score (MS) for patients with UC, and the Crohn’s Disease Activity Index (CDAI) for patients with CD, along with other inflammatory markers.

**Results:**

Of the 108 patients with IBD included in this study, 89 had UC, and 19 had CD. The NLR was significantly higher in patients with active disease than in those in remission, with *P* values of 0.011 for UC and<0.001 for CD. Additionally, there was a strong correlation between disease severity and NLR (r=0.723 for UC and r=0.887 for CD).

**Conclusion:**

The NLR is an effective marker for distinguishing between active and remissive patients with IBD and is closely associated with disease severity.

## Introduction

 Inflammatory bowel diseases (IBDs) are a range of disorders influenced by various factors, including changes in gut microbiota, immune system dysregulations, and neuropsychiatric issues, that can notably debilitate patients.^1^ Although the prevalence remains stable or declines in Western countries, studies show that these diseases are becoming more common and causing a significant burden in developing nations like Iran.^2,3^ The two main types of IBD, ulcerative colitis (UC) and Crohn’s disease (CD), are distinguished by the location of inflammation, the depth of tissue involvement, and the complications they cause.^4^

 Since CD causes transmural involvement of the gastrointestinal (GI) tract, relying solely on endoscopy is inadequate for monitoring disease activity. Therefore, it is essential to incorporate laboratory data, the clinical course, and the Crohn’s Disease Activity Index (CDAI) alongside endoscopic evaluation.^5^ The Mayo score (MS), one of the most widely used criteria for monitoring patients with UC, combines colonoscopic, clinical, and laboratory data.^6,7^ Likewise, most of the diagnostic and follow-up methods for IBDs involve colonoscopies, which are intrusive, costly, and require intestinal preparation; moreover, endoscopic findings do not always correlate with the clinical course.^8^ Thus, new markers have been proposed and examined to tackle these drawbacks. Erythrocyte sedimentation rate (ESR) and C-reactive protein (CRP) are acute-phase reactants commonly used to assess inflammation in various conditions; however, their accuracy is suboptimal in patients with IBD in previous studies.^9,10^ The levels of biomarkers such as calprotectin or lactoferrin in feces have been proposed as new diagnostic and monitoring tools; however, they are not specific and have low acceptance among patients.^11^

 The neutrophil-to-lymphocyte ratio (NLR) can be easily calculated using a simple complete blood count sampling and is being utilized as a potential marker of inflammation in numerous autoimmune, cardiovascular, metabolic, neoplastic, and infectious diseases.^12,13^ Moreover, several surveys have proposed NLR as a diagnostic and monitoring parameter for patients with IBD ^14^, as this index is notably higher in patients with IBD compared with non-IBD controls and in those with active disease compared with those in remission.^15^ Due to documented racial variations in NLR, investigations across diverse populations are beneficial.^16^ Given the limited evidence on this index among Iranian adults with IBD, this survey was conducted to contribute new data to the local and global literature. This retrospective study included patients with IBDs whose Data collected included demographics, CDAI, MS, ESR, CRP, and NLR.

## Materials and Method

###  Study population

 In this cross-sectional study, data from patients previously diagnosed with UC or CD who visited the gastroenterology clinic were retrospectively reviewed. These patients presented to Hazrat Rasool Akram Hospital between March 2022 and January 2023. The study included individuals with diagnosed IBD who were receiving treatment as necessary, with accessible demographic, laboratory, colonoscopic, and clinical records. We extracted endoscopic data and laboratory values for CRP, ESR, absolute neutrophil count (ANC), absolute lymphocyte count (ALC), and colonoscopic data from records obtained within 1 month prior to their enrollment. The NLR was calculated for each patient by dividing ANC by ALC. Patients exhibiting signs of infection, hepatic, or hematologic disorders, as well as those admitted to the emergency department, were excluded from the analysis. Additionally, patients admitted to the hospital for IBD flare-ups who received corticosteroid medications within the previous month were excluded from the study.

###  Clinical disease activity 

 Disease activity was evaluated using the MS for patients with UC and the CDAI for patients with CD. CDAI, one of the most common methods to describe disease activity in patients with CD, is used to evaluate the patient’s response to treatment and the severity of the disease.^17^ The CDAI ranges from 0 to 600, with scores below 150 indicating remission. MS, however, is used to evaluate the disease activity in patients with UC, which is invasive as it requires the patient to undergo a colonoscopy.^6^ The MS is scored on a scale from 0 to 12, with scores above 3 indicating active disease. While MS and CDAI are widely used to assess disease activity in IBD, it has been demonstrated in the literature that higher scores are associated with a more severe disease.^18,19^

###  Colonoscopy data

 Participants received instructions on bowel preparation using self-administered polyethylene glycol and dietary adjustments a day prior to their procedure. Patients were sedated in the course of the procedure using fentanyl and midazolam intravenously, supervised by an anesthesiologist. Colonoscopies were performed by two gastroenterologists and their fellows using a Fujinon Colonoscopy 2200 (Fujifilm Endoscopy; Fuji Photo Film Co., Ltd., Midtown West, Tokyo, Japan). Participants’ bowel preparations were examined and classified as good, fair, and poor. Those with poor preparation were required to undergo a second-look colonoscopy.

###  Data analysis

 The analysis was conducted using IBM SPSS Statistics software version 25.0 (IBM Corp., NY, USA) and STATA software version 15 (STATA Corp., NY, USA). The Kolmogorov-Smirnov test was initially developed to assess whether continuous variables follow a normal distribution. If the variables were found to be normally distributed, we used a parametric test, such as an independent samples t-test. If they did not follow a normal distribution, we used a non-parametric test, such as the Mann-Whitney U test. Spearman’s correlation coefficient was used to examine relationships among quantitative variables. Receiver operating characteristic (ROC) curve analysis was utilized to determine a cut-off point for NLR in active and remission patients. Additionally, a multiple linear regression was conducted to evaluate the effect of each factor on disease activity, controlling for confounding variables. In this study, the significance level of the P value was less than 0.05.

###  Ethical issues

 This survey was conducted in accordance with the Helsinki Declaration of Ethical Principles. All participants were informed about the study, and the physicians obtained written informed consent. Additionally, the research protocol received ethical approval from Iran University’s Institutional Review Board and Ethics Committee (IR.IUMS.FMD.REC.1401.567).

## Result

 This study included 108 patients with IBD, with a median time from diagnosis to study entry of 47.8 months. This included 89 patients with UC and 19 with CD. Of the total population, 18 individuals had inactive disease, and 90 had active disease, based on CDAI and MS. Of the patients entering the study, 51 (47.2%) were men, and 57 (52.8%) were women. The proportion of female patients was higher among those with inactive disease (13 females, 72.2%) than among those with active disease (44 females, 48.9%). However, this difference was not statistically significant (*P* = 0.07). The average age of the total patients was 33.42 ± 9.72, with the active group averaging 33.12 ± 10.23 and the inactive group averaging 34.89 ± 6.72. This difference was not statistically significant (*P* = 0.194). The demographic features of the patients are summarized in Table 1.

**Table 1:** Demographics of patients with active and inactive disease

**Total patients (%)**		**Average age (year)±SD**	**Male (%)**	**CD (%)**	**UC (%)**	**Months from the initial diagnosis**
Active disease	90 (83.3)	33.12 ± 10.23	46 (51.1)	11 (12.2)	79 (87.8)	45.74 ± 41.51
Inactive Disease	18 (16.6)	33.12 ± 10.23	5 (27.7)	8 (44.4)	10 (55.6)	57.78 ± 37.46
*P* value		0.194	0.07	0.003	0.003	0.096

SD: standard deviation, CD: Crohn’s disease, UC: Ulcerative colitis

 Considering disease severity, the mean ± SD for MS was 4.31 ± 3.42 in patients with UC, while the mean ± SD for the CDAI score was 259.04 ± 168.28 in patients with CD.

 During the study, patients with active UC exhibited significantly higher ESR, CRP, ANC, and NLR levels than those with silent disease (*P* < 0.05). Conversely, the ALC was marginally elevated in active patients with UC, though this difference was not statistically significant (P = 0.447). This observation was noted in patients with CD as well. Patients with active CD exhibited significantly higher levels of ESR, CRP, ANC, and NLR. In contrast, patients with inactive disease had elevated ALC levels in their peripheral blood, although this difference was not statistically significant (*P* = 0.535). Table 2 presents the data.

**Table 2:** Laboratory findings of patients

		**ESR**	**CRP**	**ANC**	**ALC**	**NLR**
UC	Active disease	16.58 ± 6.93	9.58 ± 7.09	4671.73 ± 1271.23	2293.86 ± 504.86	2.38 ± 2.29
	Inactive disease	8.80 ± 0.919	3/00 ± 1.56	3679.00 ± 772.65	2439/00 ± 487.97	1.53 ± 0.28
	*P* value	< 0.001	< 0.001	0.013	0.447	0.011
CD	Active disease	24.59 ± 6.70	14.32 ± 4.90	6438.18 ± 1348.04	2526/09 ± 658.40	2.65 ± 0.76
	Inactive disease	10.93 ± 0.99	5.50 ± 2.07	3676.25 ± 215.40	2608.75 ± 287.22	1.41 ± 0.11
	*P* value	< 0.001	< 0.001	< 0.001	0.545	< 0.001

UC: Ulcerative colitis, CD: Crohn’s disease, ESR: Erythrocyte sedimentation rate, CRP: C-reactive protein, ANC: Absolute neutrophil count, ALC: Absolute lymphocyte count, NLR: Neutrophil to lymphocyte ratio

 The study examined the correlation between inflammatory markers and disease severity, revealing that ESR, CRP, ANC, and NLR showed strong positive correlations with the severity of the disease in patients with UC, with Spearman’s coefficients recorded at 0.857, 0.801, 0.720, and 0.732, respectively. Moreover, the total ALC showed an inverse relationship with disease severity in UC patients (coefficient -0.302, *P* = 0.004). On the other hand, in CD, disease severity showed a positive correlation with ESR, CRP, ANC, and NLR (*P* < 0.001). However, more severe disease was not correlated to ALC (coefficient -0.132, *P* = 0.590). A summary of the Spearman correlation results is presented in Table 3.

**Table 3:** Spearman correlation coefficients between laboratory parameters and disease severity in patients with UC and CD

	**ESR**	**CRP**	**ANC**	**ALC**	**NLR**
UC (r) *P* value	0.857	0.801	0.720	-0.302	0.732
	< 0.001	< 0.001	< 0.001	0.004	< 0.001
CD (r) *P* value	0.875	0.908	0.805	-0.132	0.887
	< 0.001	< 0.001	< 0.001	0.590	< 0.001

UC: Ulcerative colitis, CD: Crohn’s disease, ESR: Erythrocyte sedimentation rate, CRP: C-reactive protein, ANC: Absolute neutrophil count, ALC: Absolute lymphocyte count, NLR: Neutrophil to lymphocyte ratio

 The ROC curve analysis identified 1.655 as the optimal NLR cut-off point for patients with UC, demonstrating a sensitivity of 60%, a specificity of 80%, a positive predictive value (PPV) of 27.5%, and a negative predictive value (NPV) of 94.07. Nevertheless, the area under the curve (AUC) was less than that of ESR and CRP but greater than that of ANC. This information is illustrated in Figure 1a and Table 4. In patients with CD, the NLR has the highest AUC compared with ESR, CRP, and ANC. The optimal cut-off point is 1.575, with 100% specificity, sensitivity, positive predictive value (PPV), and negative predictive value (NPV). The ROC curve for patients with CD is shown in Figure 1b and in Table 4.

**Figure 1:**
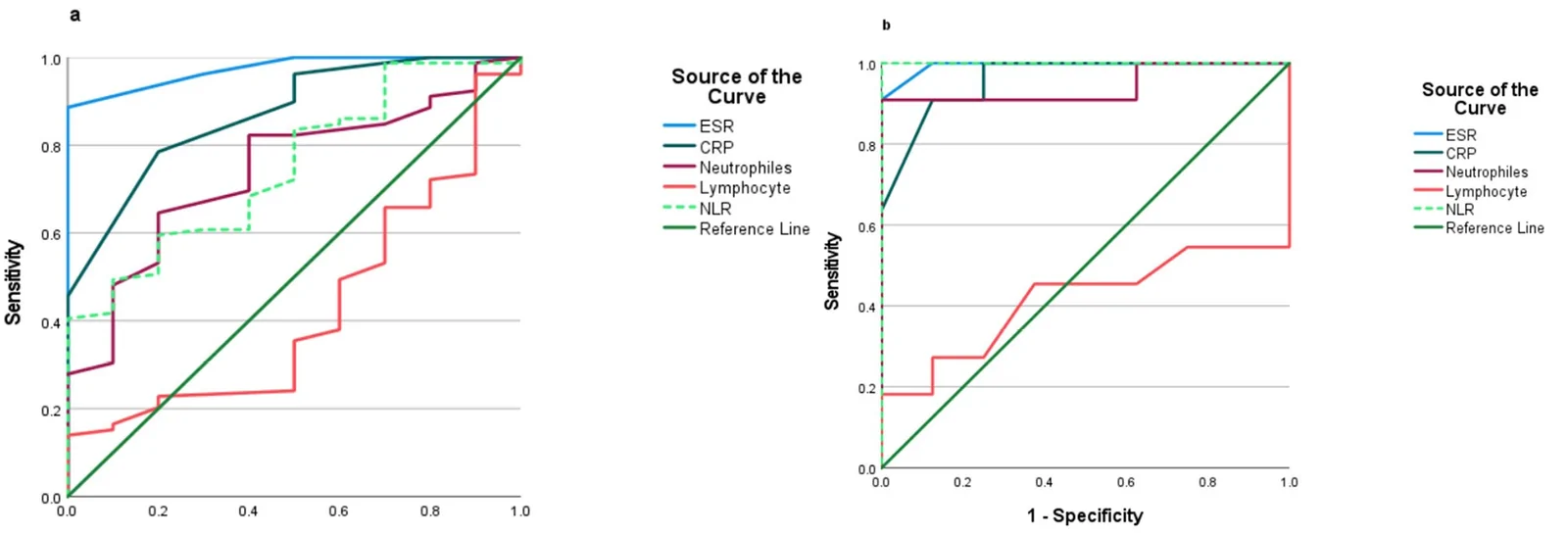
Receiver operating characteristic (ROC) curve analysis of the neutrophil-to-lymphocyte ratio (NLR) versus other inflammatory markers in predicting active disease in “a” UC patients and “b” CD patients. UC: Ulcerative colitis, CD: Crohn’s disease

**Table 4:** ROC Analysis of laboratory characteristics in distinguishing active disease

	**ESR**	**CRP**	**ANC**	**ALC**	**NLR**
UC (AUC)	0.973	0.871	0.741	0.426	0.749
CD (AUC)	0.994	0.960	0.943	0.425	1

ROC: Receiver operating characteristics, AUC: Area under the curve, UC: Ulcerative colitis, CD: Crohn’s disease, ESR: Erythrocyte sedimentation rate, CRP: C-reactive protein, ANC: Absolute neutrophil count, ALC: Absolute lymphocyte count, NLR: Neutrophil to lymphocyte ratio

 A multiple linear regression analysis was conducted to examine the relationship between activity scores and the following variables: age, sex, ESR, CRP, ANC, ALC, and NLR. The results indicated that three variables—ESR, CRP, ANC, NLR—were statistically significant correlated with activity scores in patients with UC, with results showing F (8, 77) = 57.60, *P* < .0001, R^2^ = .857. On the other hand, age (*P* = 0.28), sex (*P* = 0.62), and ALC (*P* = 0.16) did not show significant associations. Additionally, none of the variables demonstrated a significant association with the CDAI. These data are demonstrated in Table 5. The lack of a statistically significant relationship between the measured factors and disease severity in CD could be attributed to the relatively small number of patients with CD, as well as collinearity among the factors, including ESR, CRP, ANC, ALC, and NLR. However, the variance inflation factor (VIF) was evaluated in this context and was greater than one but less than ten.

**Table 5:** Multivariate linear regression between measured factors and disease activity

**UC (MS)**					**CD (CDAI)**	
	**Coefficient Std.**	**Sig. **	**95% confidence** ** interval**	**Coefficient Std.**	**Sig. **	**95% confidence interval**
Age	-0.0199	0.173	-0.0487 - 0.0089	-1.730	0.520	-7.57 - 4.118
Sex	0.128	0.659	-0.4486 - 0.7055	-2.728	0.946	-91.22 - 85.76
ESR	0.122	0.041	0.0050 -0.2392	1.754	0.799	-13.376 - 16.88
CRP	0.223	0.0001	0.1133 - 0.3336	2.027	0.767	-12.990 - 17.046
ANC	0.0006	0.008	0.0001 - 0.0010	0.1275	0.090	-0.02441 - 0.279
ALC	0.0005	0.152	-0.0012 - 0.0001	-0.183	0.187	-0.4733 - 0.1067
NLR	0.098	0.042	0.0038 - 0.2415	-117.762	0.245	-331.84 - 96.32

UC: Ulcerative colitis, CD: Crohn’s disease, ESR: Erythrocyte sedimentation rate, CRP: C-reactive protein, ANC: Absolute neutrophil count, ALC: Absolute lymphocyte count, NLR: Neutrophil to lymphocyte ratio, MS: Mayo Score, CDAI: Crohn’s disease Activity index

## Discussion

 Our study aimed to evaluate the value of NLR as an emerging non-invasive marker indicating disease severity and activity in the Iranian adult population with IBD. The results revealed that ESR, CRP, NLR, and ANC levels were higher in patients with active UC and CD. Moreover, these markers showed a strong correlation with disease severity. Conversely, ALC did not show a statistically significant difference between patients with active and inactive disease in UC or CD, although a weak negative correlation between ALC and disease severity was observed in patients with UC; this association lost significance after adjustment for other variables. In patients with CD, NLR was a more sensitive and specific marker for diagnosing active disease than ESR, CRP, and ANC; however, in patients with UC, ESR and CRP were more valuable in this regard, despite no significant correlation between the variables and CD severity in linear regression.

 Considering previous studies on this issue, in the meta-analysis conducted by Wei and colleagues, the non-invasive NLR method effectively distinguishes between patients with active UC and those in remission, yielding a standard mean difference (SMD) of 1.32. Furthermore, the SMD for differentiating patients with active CD from those in remission was 1.91 (*P* < 0.05).

 Moreover, Langley and others identified eight studies examining inflammatory markers in their systematic scoping review, only one of which found no difference in NLR between active and inactive CD.^12^ In a previous study by Gao and co-workers, NLR had the highest AUC (0.854) for distinguishing between CD patients and healthy individuals, compared with ESR, CRP, and total white blood cell count. However, the NLR did not show a statistically significant ability to differentiate between active and inactive forms of CD.^20^

 As previously noted, these studies have been conducted in certain countries, including Turkey, the United States, China, Korea, and India, where the results are inconsistent in some instances.^20,21,22,23^ In the only Indian study conducted to date on NLR among patients with varying severities of IBD, a significant difference was observed between both UC and CD patients (*P* < 0.001).^24^ Furthermore, eight studies conducted in Turkey indicated that the SMD between active and remission of CD was higher than the active and remission of UC, 2.35 (1.81-2.89) and 1.18 (0.67-1.69), respectively.^21^ This was consistent with our results, which indicated that NLR could be a more valuable marker in CD than in UC. Remarkably, two studies from China found no substantial difference in NLR between patients with active and remissive CD.^20,25^ The remaining studies from other countries predominantly focused on patients with UC and demonstrated the tendency of NLR in differentiating patients with UC from healthy controls and those with silent disease.^9,15^ Furthermore, we have identified only one study in Iran on NLR in patients with IBD that assesses pediatric patients with IBD. In this study, NLR was higher in patients with active disease compared with those in remission, although this difference was not statistically significant (*P* = 0.056), suggesting differences between pediatric and adult IBD.^26,27^

 The current study identified the strongest correlation between disease severity and CRP levels in patients with UC. This finding aligns with the results of studies conducted by Demir and colleagues in Turkey and Okba and others in Egypt. In the latter study, CRP was the only parameter that effectively distinguished between active and remissive stages of UC. Moreover, in both studies, CRP exhibited the highest AUC when comparing active and inactive UC.^28,29^ These findings suggest that NLR cannot be used in isolation and should be considered alongside other laboratory factors.

 Conversely, we discovered that NLR was a more reliable indicator than CRP for identifying active CD. Nonetheless, past research from China and Turkey contradicts our finding, suggesting that CRP is a more significant metric.^21,22^ Additionally, the optimal cut-off points we measured for CD and UC were lower than in previous studies.^15,21^ Unlike the studies mentioned earlier, we found no statistically significant difference between ALC and disease severity.^4,30^ The discrepancy may be attributed to differences in medication or ethnic factors of our study participants. Moreover, there are far fewer studies focused on CD compared with UC.^12,16^

 Our study appears to be unique, as it is the only research on the Iranian adult population regarding IBD, specifically UC and CD. We measured cut-off points for active disease using the most recent and widely accepted scoring systems. However, we acknowledge some limitations in our study, including a relatively small sample size, especially among patients with CD, the cross-sectional design, and the lack of data on patients’ current treatments. These factors underscore the necessity for further research in this area. Despite these limitations, our findings demonstrate the value of the non-invasive NLR alongside other non-invasive parameters for evaluating disease activity and severity in patients with IBD.

## Conclusion

 The NLR can effectively differentiate between active and remissive Iranian patients with IBD and is closely associated with disease severity. Although conventional inflammatory markers like CRP are particularly useful for UC, the NLR appears to be the most valuable indicator for CD. Nonetheless, additional cohort and matched-group studies are essential to further explore this issue.

## References

[1] Tavakoli P, Vollmer-Conna U, Hadzi-Pavlovic D, Grimm MC (2021). A Review of Inflammatory Bowel Disease: A Model of Microbial, Immune and Neuropsychological Integration. Public Health Rev.

[2] Eltantawy N, El-Zayyadi IAE-H, Elberry AA, Salah LM, Abdelrahim MEA, Kassem AB (2023). A review article of inflammatory bowel disease treatment and pharmacogenomics. Beni-Suef University Journal of Basic and Applied Sciences.

[3] Malekzadeh MM, Vahedi H, Gohari K, Mehdipour P, Sepanlou SG, Ebrahimi Daryani N (2016). Emerging Epidemic of Inflammatory Bowel Disease in a Middle Income Country: A Nation-wide Study from Iran. Arch Iran Med.

[4] Acarturk G, Acay A, Demir K, Ulu MS, Ahsen A, Yuksel S (2015). Neutrophil-to-lymphocyte ratio in inflammatory bowel disease - as a new predictor of disease severity. Bratisl Lek Listy.

[5] Lo Re G, Cappello M, Tudisca C, Galia M, Randazzo C, Craxì A (2014). CT enterography as a powerful tool for the evaluation of inflammatory activity in Crohn’s disease: relationship of CT findings with CDAI and acute-phase reactants. Radiol Med.

[6] Lewis JD, Chuai S, Nessel L, Lichtenstein GR, Aberra FN, Ellenberg JH (2008). Use of the noninvasive components of the Mayo score to assess clinical response in ulcerative colitis. Inflamm Bowel Dis.

[7] Naegeli AN, Hunter T, Dong Y, Hoskin B, Middleton-Dalby C, Hetherington J (2021). Full, Partial, and Modified Permutations of the Mayo Score: Characterizing Clinical and Patient-Reported Outcomes in Ulcerative Colitis Patients. Crohns Colitis 360.

[8] Cui J, Li X, Zhang Z, Gao H, Li J (2022). Common laboratory blood test immune panel markers are useful for grading ulcerative colitis endoscopic severity. BMC Gastroenterol.

[9] Lin H, Bai Z, Wu Q, Chu G, Zhang Y, Guo X (2022). Inflammatory Indexes for Assessing the Severity and Disease Progression of Ulcerative Colitis: A Single-Center Retrospective Study. Front Public Health.

[10] Menees SB, Powell C, Kurlander J, Goel A, Chey WD (2015). A meta-analysis of the utility of C-reactive protein, erythrocyte sedimentation rate, fecal calprotectin, and fecal lactoferrin to exclude inflammatory bowel disease in adults with IBS. Am J Gastroenterol.

[11] Lehmann FS, Burri E, Beglinger C (2015). The role and utility of faecal markers in inflammatory bowel disease. Ther Adv Gastroenterol.

[12] Langley BO, Guedry SE, Goldenberg JZ, Hanes DA, Beardsley JA, Ryan JJ (2021). Inflammatory Bowel Disease and Neutrophil-Lymphocyte Ratio: A Systematic Scoping Review. J Clin Med.

[13] Shadmanfar S, Masoumi M, Davatchi F, Shahram F, Akhlaghi M, Faezi ST (2021). Correlation of clinical signs and symptoms of Behçet’s disease with platelet-to-lymphocyte ratio (PLR) and neutrophil-to-lymphocyte ratio (NLR). Immunol Res.

[14] Feng W, Liu Y, Zhu L, Xu L, Shen H (2022). Evaluation of neutrophil-to-lymphocyte ratio and platelet-to-lymphocyte ratio as potential markers for ulcerative colitis: a retrospective study. BMC Gastroenterol.

[15] Ma L, Pang X, Ji G, Ma X, Li J, Chang Y (2021). Application of the neutrophil to lymphocyte ratio in the diagnosis and activity determination of ulcerative colitis: A meta-analysis and systematic review. Medicine (Baltimore).

[16] Azab B, Camacho-Rivera M, Taioli E (2014). Average values and racial differences of neutrophil lymphocyte ratio among a nationally representative sample of United States subjects. PLoS One.

[17] Hutchings H, Alrubaiy L. Crohn’s Disease Activity Index, in Encyclopedia of Quality of Life and Well-Being Research, A.C. Michalos, Editor. 2014, Springer Netherlands: Dordrecht. p: 1354-7. doi: 10.1007/978-3-031-17299-1_621.

[18] Pabla BS, Schwartz DA (2020). Assessing Severity of Disease in Patients with Ulcerative Colitis. Gastroenterol Clin North Am.

[19] Shaoul R, Day AS (2021). An Overview of Tools to Score Severity in Pediatric Inflammatory Bowel Disease. Front Pediatr.

[20] Gao SQ, Huang LD, Dai RJ, Chen DD, Hu WJ, Shan YF (2015). Neutrophil-lymphocyte ratio: a controversial marker in predicting Crohn’s disease severity. Int J Clin Exp Pathol.

[21] Fu W, Fu H, Ye W, Han Y, Liu X, Zhu S (2021). Peripheral blood neutrophil-to-lymphocyte ratio in inflammatory bowel disease and disease activity: A meta-analysis. Int Immunopharmacol.

[22] Jeong Y, Jeon SR, Kim HG, Moon JR, Lee TH, Jang JY (2021). The role of platelet to lymphocyte ratio and neutrophil to lymphocyte ratio in ulcerative colitis. Intest Res.

[23] Zhang MH, Wang H, Wang HG, Wen X, Yang XZ (2021). Effective immune-inﬂammation index for ulcerative colitis and activity assessments. World J Clin Cases.

[24] Patel DM, Patel DK, Patel MM, Patel LB, Shaikh RT, Shaikh NT (2025). Neutrophil-lymphocyte, Lymphocyte-monocyte, and Platelet-lymphocyte Ratios: Non-invasive Biomarkers for Assessing Inflammatory Bowel Disease Activity. Recent Adv Inflamm Allergy Drug Discov.

[25] Feng JR, Qiu X, Wang F, Chen PF, Gao Q, Peng YN (2017). Diagnostic Value of Neutrophil-to-Lymphocyte Ratio and Platelet-to-Lymphocyte Ratio in Crohn’s Disease. Gastroenterol Res Pract.

[26] Zahmatkesh A, Sohouli MH, Hosseini SME, Rohani P (2023). The role of platelet-to-lymphocyte ratio and neutrophil-to-lymphocyte ratio in the diagnosis and severity of inflammatory bowel disease in children. Eur J Pediatr.

[27] Kammermeier J, Lamb CA, Jones KDJ, Anderson CA, Baple EL, Bolton C (2023). Genomic diagnosis and care co-ordination for monogenic inflammatory bowel disease in children and adults: consensus guideline on behalf of the British Society of Gastroenterology and British Society of Paediatric Gastroenterology, Hepatology and Nutrition. Lancet Gastroenterol Hepatol.

[28] Demir AK, Demirtas A, Kaya SU, Tastan I, Butun I, Sagcan M (2015). The relationship between the neutrophil-lymphocyte ratio and disease activity in patients with ulcerative colitis. Kaohsiung J Med Sci.

[29] Okba AM, Amin MM, Abdelmoaty AS, Ebada HE, Kamel AH, Allam AS (2019). Neutrophil/lymphocyte ratio and lymphocyte/monocyte ratio in ulcerative colitis as non-invasive biomarkers of disease activity and severity. Auto Immun Highlights.

[30] Torun S, Tunc BD, Suvak B, Yildiz H, Tas A, Sayilir A (2012). Assessment of neutrophil-lymphocyte ratio in ulcerative colitis: a promising marker in predicting disease severity. Clin Res Hepatol Gastroenterol.

